# Glycated hemoglobin (HbA1c) is independently associated with the bioelectrical impedance phase angle in junior sumo wrestlers: A pilot study

**DOI:** 10.14814/phy2.16045

**Published:** 2024-05-13

**Authors:** Suraiya Khatun, Miori Ogawa, Akiko Uchizawa, Daisuke Hoshi, Shinsuke Tamai, Reiko Momma, Emi Kondo, Koichi Watanabe, Hiroyuki Sagayama

**Affiliations:** ^1^ Doctoral Program in Sports Medicine, Graduate School of Comprehensive Human Sciences University of Tsukuba Ibaraki Japan; ^2^ Department of Pediatrics Japanese Red Cross Nasu Hospital Tochigi Japan; ^3^ Institute of Health and Sports Sciences University of Tsukuba Ibaraki Japan; ^4^ Japan Society for the Promotion of Science Tokyo Japan; ^5^ Human Informatics and Interaction Research Institute National Institute of Advanced Industrial Science and Technology Tsukuba Ibaraki Japan; ^6^ Department of Sport Science and Research Japan Institute of Sports Sciences Tokyo Japan; ^7^ Advanced Research Initiative for Human High Performance (ARIHHP) University of Tsukuba Tsukuba Japan

**Keywords:** bioelectrical impedance analysis, glycated hemoglobin, metabolic parameters, phase angle

## Abstract

The study explores the relationship between phase angle (PhA), an indicator of cellular health, and metabolic health parameters among junior sumo wrestlers in Japan. Given the demanding lifestyle and high‐energy diets of sumo wrestlers that predispose them to metabolic syndrome post‐retirement, this study focuses on a younger cohort. The primary aim is to evaluate if PhA could serve as an early indicator of metabolic health issues within this unique demographic. A total of 14 sumo wrestlers aged 9–17 years were assessed to determine the relationship between PhA and various metabolic markers, including glycated hemoglobin (HbA1c), using a TANITA MC‐780A‐N body composition analyzer and standard blood tests. Bivariate regression analysis and Pearson's correlation revealed a negative relationship between PhA and HbA1c even after adjusting for age and weight (*ß* = −0.496, *r* ^2^ = 0.776, *r* = −0.756, *p* = 0.004). The results indicate a significant negative relationship between PhA and HbA1c levels, suggesting that lower PhA values, which indicate poorer cellular integrity, are associated with higher HbA1c levels, signifying impaired glycemic control. These findings underscore the potential of PhA as a valuable biomarker for monitoring metabolic health in young sumo wrestlers, with implications for early intervention and management strategies.

## 1 INTRODUCTION

Japan has a rich history of sports; among its most traditional and esteemed sports is sumo wrestling, which holds the status of a national sport. Sumo wrestlers dedicate their lives to this sport, adhering to strict regulations set by the Japan Sumo Association (Kanda et al., 2008). Their daily routine involves rigorous training and a high‐energy diet, with some consuming more than 10,000 kcal/day (Nishizawa, Akaoka, Nishida, Kawaguchi, & Hayashi, 1976). Most sumo wrestlers lead their lifestyle with repeated high energy intake from childhood, and they have to maintain their large body structure for as long as they are associated with this sport. However, a high caloric diet is associated with an increase in waist circumference (WC), blood glucose, triglyceride (TG) concentration, and other metabolic parameters (Casazza et al., 2009). Such demanding lifestyle, characterized by the consumption of a high‐energy diet and daily engagement in high‐intensity interval training, may take a toll on the health of sumo wrestlers; indeed, studies have shown that the life expectancy of sumo wrestlers is typically 10–12 years lower than that of the average Japanese person (Saito et al., 2003). Moreover, upon retirement from sports, sumo wrestlers are affected by metabolic syndromes (MetS), such as stroke, diabetes, and heart problems (Nishizawa, Akaoka, Nishida, Kawaguchi, & Hayashi, 1976).

MetS is a medical term used to describe diabetes, insulin resistance, high blood pressure (BP), cardiovascular disease, and stroke (Alberti et al., 2009). Several factors are involved in the development of MetS, but the most crucial ones are obesity, visceral fat (causing insulin resistance), and a sedentary lifestyle (Alberti et al., 2009; Kaur, 2014; Kelishadi, 2007). MetS is associated with the following metabolic factors: a rise in BP; an increase in fasting blood glucose, low‐density lipoprotein cholesterol (LDL‐C), TG, and WC; and a decrease in high‐density lipoprotein cholesterol (HDL‐C) (Panda, 2019). In a previous study (Ogawa et al., 2021), almost no differences were found in the levels of metabolic parameters between junior sumo wrestlers and other sports groups of children, except for WC (higher than other sports groups), HDL‐C (lower than other sports groups), and systolic blood pressure (SBP) (higher than other sports groups). However, this previous study (Ogawa et al., 2021) did not measure the phase angle (PhA) value nor did it explore the potential relationship between PhA and metabolic parameters within this cohort of children. PhA has been used as a predictive biomarker for cellular health and integrity, and it is an early sign of clinical complications (Barrea et al., 2019). PhA is calculated from raw bioelectrical impedance parameters, that is, resistance (*R*) and reactance (*X*c) (Sacco et al., 2021). The human body consists of conductive (biological fluids) and non‐conductive (fat and bone) components. Various types of ions present in biological fluids (blood, plasma, saliva, and mucus) facilitate the flow of an administered alternating current (AC). When the flow of this AC is altered due to viscosity and the presence of different substances, this is termed electrical *R*. Additionally, when an AC flow is administered to the body using bioelectrical impedance analysis (BIA), the tissue cell membranes charge themselves by arresting the electrical current, resulting in a delay in the electrical flow; this is known as the capacitance property of the cell membrane, also called *X*c. The *R* and *X*c represent the impedance (*Z*) [*Z* ^2^ = *R* ^2^ + *X*c (Nishizawa, Akaoka, Nishida, Kawaguchi, & Hayashi, 1976)], and PhA expresses the position of the impedance vector. Because of the presence of these capacitors (cell membranes), the current leads the voltage, or the voltage lags the current, resulting in the formation of a negative PhA (delay) value (Foster & Lukaski, 1996; Lukaski, 1996, 2013). The normal PhA value ranges between about 5° and 12° in healthy and physically active participants (Norman et al., 2012). PhA can also serve as a useful predictor for the intracellular water (ICW) pool, the ratio of extracellular water (ECW) to ICW (ECW/ICW), and the cellular hydration status (Francisco et al., 2020). A higher PhA value predicts a larger ICW pool and a lower ECW/ICW ratio, which are both indicative of a healthier cellular membrane and better cell function, as ICW is contained within cells where metabolic processes occur. Conversely, a lower PhA value is associated with an increased ECW/ICW ratio, suggesting potential cell membrane compromise or fluid imbalance, such as overhydration or inflammation.

However, the PhA value does not always remain steady. Rather, it decreases repeatedly compared to the normal range due to a decrease in the capacitive properties of the cell membrane resulting from different kinds of diseases, such as kidney disease (Zdolsek et al., 2000), viral and microbial infections such as HIV (Norman et al., 2012) and pulmonary tuberculosis (Kumar et al., 2014), and different types of cancers such as pancreatic (Gupta, Lis, et al., 2004), colorectal (Gupta et al., 2008; Gupta, Lammersfeld, et al., 2004), and lung (Gupta et al., 2009) cancer. The PhA value also decreases due to an increase in the levels of metabolic parameters such as cholesterol, insulin, glucose, homeostatic assessment for insulin resistance (HOMA‐IR), and leptin (de Luis et al., 2010). In addition, PhA is inversely correlated with WC in adults (Longo et al., 2021), and with glycated hemoglobin (HbA1c) in type 2 diabetes (T2D) patients (Choi et al., 2020; Dittmar et al., 2015). Age (PhA decreases with age), sex (males have greater PhA values than females) (Cristina et al., 2005), and lifestyle (Mundstock et al., 2019) are also involved in PhA alterations. In the case of obese individuals, PhA may initially increase with body mass index (BMI) up to a certain level of obesity (up to 35 kg/m^2^), and then decrease as BMI continues to rise (>35 kg/m^2^). Specifically, obesity may be associated with a higher variability of the *R* and *X*c parameters that determine the PhA value, due to the variation in fat and fat‐free mass (Cancello et al., 2023). The *R* and *X*c values are measured using a bioelectrical impedance device; these are also known as the raw bioelectrical impedance parameters, and they are used to calculate PhA with the following formula: [arc tangent (*X*c/*R*) × 180°/π] (Sacco et al., 2021). According to Asmasary et al. (2023), the mean HbA1c level in obese children is reported to be 3.59% (ranging from 2% to 6.2%), whereas in non‐obese children 3.65% (ranging from 1.9% to 5.3%). However, the mean HbA1c level among our study's subjects was 5.6% (ranging from 5.4% to 5.9%). Vijayakumar et al. (Vijayakumar et al., 2017) reported that individuals with consistently high HbA1c levels (5.7% or above) are at an increased risk of developing diabetes. In their study, they examined 2095 children aged 10–19 years monitored through the age of 39, and 2005 adults aged 20–39 years monitored through the age of 59, and categorized HbA1c levels into three groups: prediabetes (5.7%–6.4%), intermediate (5.4%–5.6%), and lower (5.3% or below). The participants in our study had HbA1c levels ranging from 5.4% to 5.9%, placing even the lowest measured levels within the intermediate category.

In this study, we hypothesized that alterations in metabolic parameters and HbA1c levels are associated with variations in PhA, which could explain an individual's health condition by increasing or decreasing the degree of PhA. This hypothesis was supported by findings from our previous study (Ogawa et al., 2021), which demonstrated significant differences in metabolic parameters, including WC, obesity rate, BMI, SBP, and HDL‐C compared to other sports groups of children. Some studies have shown a considerable association between bioelectrical impedance PhA and metabolic parameters, including HbA1c in adults (Barrea et al., 2016; Choi et al., 2020; Dittmar et al., 2015; Longo et al., 2021; Moreto et al., 2017); however, no study has shown this association in junior sumo wrestlers who despite their young age and physical activity have a higher body mass than normal children. In this study, we used junior sumo wrestlers to measure early signs of MetS and glycemic control using bioelectrical impedance PhA. The selection of sumo children as study subjects is based on their unique physiological and lifestyle characteristics related to sumo wrestling. Sumo wrestlers need to consume a high‐energy diet to maintain a large body structure. As a result, they are more prone to be affected by MetS than other athletes, making them an ideal group for examining the impact of bioelectrical impedance PhA on metabolic parameters. Therefore, this study aimed to clarify the association between PhA measured using BIA and metabolic parameters, including HbA1c, in junior sumo wrestlers.

## 2 MATERIALS AND METHODS

Fourteen junior male sumo wrestlers with an average age of 14.3 ± 2.4 years, weight of 95.3 ± 20.6 kg, and BMI of 33.4 ± 7.6 kg/m^2^ were enrolled. According to the questionnaire report, they were free from MetS, such as diabetes, hypertension, etc. The participants were recruited from a local sumo wrestling club, and all of them engaged in the same amount of training (approximately 3 h/day, 6 days/week). This group included nine certified prefectural athletes proficient in participating in national tournaments in Japan. All physical parameters (anthropometric measurements) and fasting venous blood samples were collected after overnight fasting, at least 24 h after the last exercise session at the sumo training center.

### 2.1 Measurement of bioelectrical impedance phase angle (PhA)

The *R* and *X*c values were measured using a multifrequency segmental body composition analyzer TANITA MC‐780A‐N (TANITA Co., Ltd., Tokyo, Japan), which is a widely recognized instrument for the measurement of body composition and PhA. Our BIA device demonstrated the accuracy of FFM variables with a concordance correlation coefficient of 0.922, precision of 0.962, and accuracy of 0.959 compared with DXA (*n* = 19, young males). Moreover, the device showed reliability as the intraclass correlation coefficient and coefficient of variation for FFM were 0.993 and 0.50, respectively, on two consecutive days (*n* = 13, young males). This advanced analyzer utilizes three different frequencies (5, 50, and 250 kHz) to enhance the measurement accuracy. The values of *R*, Xc, and PhA were reported at 50 kHz for comparison with previous studies. To ensure a comprehensive assessment, we recorded the average PhA values obtained from the left and right sides of the body.

### 2.2 Anthropometric measurements

For anthropometric measurements, height was assessed using a stadiometer (DST‐210S, Muratec‐KDS Corp., Kyoto, Japan) and body weight was recorded using a digital scale (MC‐780A‐N, TANITA‐Tokyo, Japan). WC was measured in a standing position at the level of the navel using a non‐stretchable measuring tape (SP‐715; Sekisui Jushi Corp., Osaka, Japan). To determine the BMI, the weight in kilograms was divided by the square of the height in meters, that is, body weight (kg)/height (m^2^).

### 2.3 Blood pressure and blood biomarkers

BP was measured in the sitting position using an automated BP monitor (Omron HBP‐1300, Omron Corp., Kyoto, Japan), a well‐known device for measuring BP. A study by Meng et al. (2016) reported that the average discrepancy between manual readings by professional doctors and those obtained with the Omron HBP‐1300 was within acceptable limits: 1.4 ± 3.2 mmHg for SBP and 1.0 ± 3.9 mmHg for diastolic blood pressure (DBP); that is, the device's SBP readings have an average deviation of approximately 1.4 mmHg with a standard error margin of ±3.2 mmHg, and for DBP the average deviation is around 1.0 mmHg with a standard error margin of ±3.9 mmHg. These findings are consistent in both children and adults. The following metabolic parameters were measured from fasting venous blood samples: lipid profile, including total cholesterol (enzymatic method), HDL‐C and LDL‐C (direct‐enzymatic method), and non‐HDL‐C; and glucose tolerance indicators, such as blood glucose (immobilized enzyme electrode method) and HbA1c (HPLC method). Blood samples were analyzed at local clinical laboratories (Tsukuba i‐Laboratory LLP, Ibaraki, Japan).

### 2.4 Statistical analysis

Descriptive statistical analyses, including mean and standard deviation, were performed for all variables. The normal distribution of the data was assessed using the Kolmogorov–Smirnov and Shapiro–Wilk tests (*p* > 0.05 for normal distribution), as well as by examining skewness and kurtosis coefficients and graphical analysis. Except for PhA (K–S test, *p* = 0.008; S–W test, *p* = 0.052) and TG (K–S test, *p* < 0.001; S–W test, *p* = 0.002), all other data were normally distributed. To handle the non‐normal distribution of PhA, logarithmic transformation was applied to improve normality (K–S and S–W test, *p* = 0.142 and *p* = 0.399, respectively). However, the TG data were not normally distributed. Linear regression models (crude and adjusted) were used to determine the association among PhA (dependent variable), metabolic parameters, and HbA1c (independent variables). Pearson's and Spearman's correlation coefficients were used to measure the strength of the association among PhA, metabolic profiles, and HbA1c. All statistical analyses were performed using IBM SPSS version 29.

## 3 RESULTS

**FIGURE 1:**
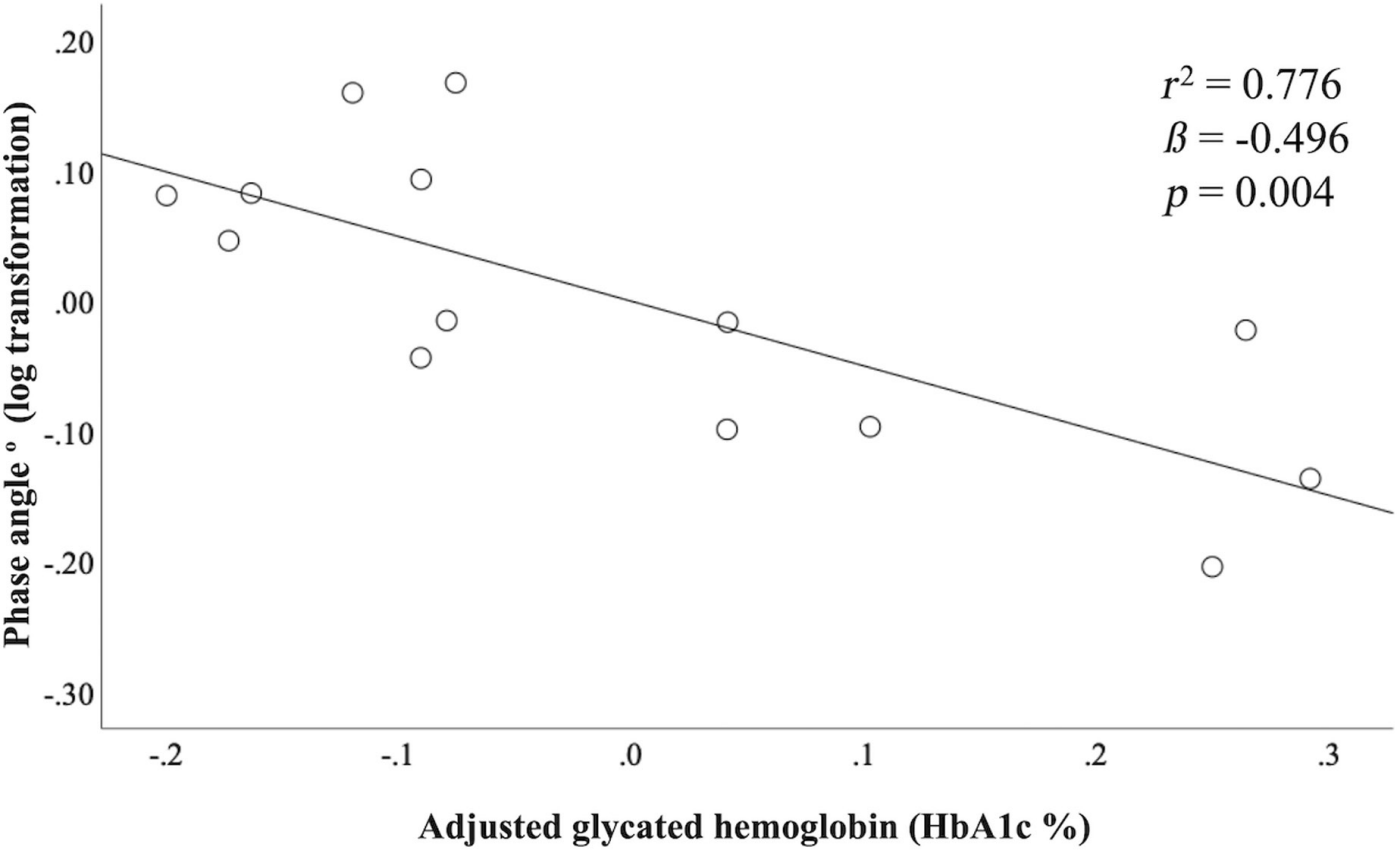
Relationship between phase angle (PhA) and glycated hemoglobin (HbA1c %). Hierarchical regression plot adjusted for age and weight, showing an inverse association between phase angle and glycated hemoglobin (HbA1c %; *r* ^2^ = 0.776, *ß* = −0.496, *p* = 0.004).

Descriptive statistics were used to present the means and standard deviations (Table 1) of all variables. To assess the association between PhA, which served as the outcome variable, and metabolic parameters including HbA1c, a simple linear regression model was applied. Bivariate regression analysis (Table 2) revealed a negative association between PhA and WC (*ß* = −0.006, *r* ^2^ = 0.324, *p* = 0.034) and HbA1c (*ß* = −0.541, *r* ^2^ = 0.413, *p* = 0.013). However, when adjusting for age and weight using hierarchical regression analysis (Table 2; Figure 1), only HbA1c showed a significant negative association with PhA (*ß* = −0.496, *r* ^2^ = 0.776, *p* = 0.004). After adjusting for age and weight, WC and PhA showed a negative association (*ß* = −0.008) but were not statistically significant (*p* = 0.453). Pearson's or Spearman's correlation coefficient analyses were used to measure the strength of the association between PhA and metabolic parameters, including HbA1c levels. PhA and HbA1c levels showed a strong negative correlation (*r* = −0.756, *p* = 0.004) (Table 3), even after adjusting for age and weight.

**TABLE 1:** Characteristics of participants.

Variables	Mean	SD
Age (years)	14.3	2.4
Height (cm)	168.4	7.6
Weight (kg)	95.3	20.6
BMI (kg/m^2^)	33.4	5.9
Waist circumference (cm)	102	13.5
SBP (mmHg)	136	9.6
DBP (mmHg)	68.7	5.8
HDL‐C (mg/dL)	44.9	4.3
LDL‐C (mg/dL)	101.1	29.9
Non‐HDL‐C (mg/dL)	114.1	36.9
Triglycerides (mg/dL)	110	77.1
Blood glucose (mg/dL)	95.6	4.9
HbA1c (%)	5.6	0.2
Total cholesterol (mg/dL)	158.8	38.6
Phase angle (°)	6.8	0.7

Abbreviations: BMI, body mass index; DBP, diastolic blood pressure; HDL, high‐density lipoprotein; LDL, low‐density lipoprotein; PhA, phase angle; SBP, systolic blood pressure; TC, total cholesterol; TG, triglycerides, HbA1c, glycated hemoglobin; WC, waist circumference.

**TABLE 2:** Crude and adjusted linear regression.

Variables	Simple linear regression			Adjusted linear regression		
Phase angle (°)	*ß*	*r* ^2^	*p*	*ß*	*r* ^2^	*p*
WC (cm)	−**0**.006*	0.324	**0**.**034**	−0.008	0.506	0.453
SBP (mmHg)	−0.001	0.006	0.794	0.005	0.545	0.246
DBP (mmHg)	−0.005	0.031	0.547	0.004	0.495	0.558
HDL‐C (mg/dL)	−0.003	0.010	0.740	0.004	0.491	0.605
LDL‐C (mg/dL)	0.000	0.001	0.907	0.002	0.560	0.199
Non‐HDL‐C (mg/dL)	0.000	0.010	0.738	0.001	0.572	0.164
TG (mg/dL)	0.000	0.039	0.497	0.001	0.596	0.116
Blood glucose (mg/dl)	−0.001	0.001	0.928	0.000	0.476	0.962
TC (mg/dL)	0.000	0.007	0.783	0.001	0.572	0.165
HbA1c (%)	−**0**.541*	0.413	**0**.**013**	−**0**.496**	0.776	**0**.**004**

*Note*: Adjusted with age and weight **p < 0.01 *p < 0.05 in bold values.

Abbreviations: BMI, body mass index; DBP, diastolic blood pressure; HDL, high‐density lipoprotein; LDL, low‐density lipoprotein; PhA, phase angle; SBP, systolic blood pressure; TC, total cholesterol; TG, triglycerides, HbA1c, glycated hemoglobin; WC, waist circumference.

**TABLE 3:** Pearson's (Spearman's correlation in case TG) and partial correlation between PhA and metabolic parameters including HbA1c.

Variables	Correlation coefficient		Partial correlation coefficient	
Phase angle (°)	*r*	*p*	*r*	*p*
WC (cm)	−**0**.**569**	**0**.**034**	−0.240	0.453
SBP (mmHg)	−0.077	0.794	0.363	0.246
DBP (mmHg)	−0.176	0.547	0.188	0.558
HDL‐C (mg/dL)	−0.098	0.740	0.167	0.605
LDL‐C (mg/dL)	0.034	0.907	0.399	0.199
Non‐HDL‐C (mg/dL)	0.098	0.738	0.429	0.164
TG (mg/dL)	−0.303	0.292	−0.334	0.289
Blood glucose (mg/dL)	−0.027	0.928	−0.015	0.962
TC (mg/dL)	0.081	0.783	0.428	0.165
HbA1c (%)	−**0**.**642**	**0**.**013**	−**0**.**756**	**0**.**004**

*Note*: Partial correlation in control of age and weight *p* < 0.05 in bold values. PhA: Outcome variable.

Abbreviations: BMI, body mass index; DBP, diastolic blood pressure; HDL, high‐density lipoprotein; LDL, low‐density lipoprotein; PhA, phase angle; SBP, systolic blood pressure; TC, total cholesterol; TG, triglycerides, HbA1c, glycated hemoglobin; WC, waist circumference.

## 4 DISCUSSION

This study aimed to explore the relationship between PhA and various metabolic parameters, including HbA1c levels, in a specific group of junior sumo wrestlers. The findings of this study revealed a robust and noteworthy negative correlation between PhA and HbA1c (Figure 1) even after adjusting for age and weight. This implies that higher HbA1c levels are associated with lower PhA in this specific group. This inverse relationship between PhA and HbA1c highlights a potential connection between glycemic control and body composition in junior sumo wrestlers. In contrast, after adjusting for age and body weight, PhA did not exhibit a significant association with other metabolic parameters, especially WC (before adjustment WC showed a significant relationship with PhA). However, before adjusting for age and weight, WC was negatively associated with PhA. This relationship suggests that PhA can be used as a clinical indicator of alterations in metabolic parameters, including HbA1c levels, in sumo children.

The measurement of PhA is a simple method that uses the *R* and *X*c parameters acquired from 50 kHz AC current via a BIA device, and this PhA value is calculated using the following formula: [arc tangent (Xc/R) × 180/π] (Foster & Lukaski, 1996; Lukaski, 1996, 2013; Sacco et al., 2021). PhA exhibits a decline in value under certain health conditions, including MetS, cancer, and viral infections. These diseases are associated with damage to the body's cells, causing a decrease in the capacity of cells to capture electrical energy (Dittmar et al., 2015; Gupta et al., 2008; Gupta, Lammersfeld, et al., 2004; Gupta, Lis, et al., 2004; Kumar et al., 2014; Norman et al., 2012; Zdolsek et al., 2000). According to a previous study (Ogawa et al., 2021) in the junior sumo group, HDL‐C, TG, and blood glucose levels were lower, but SBP was higher than the reference values. However, no substantial difference was observed in HbA1c levels. Yoshinaga et al. (2005) revealed that increased insulin levels and WC in Japanese girls and boys contributed to an increase in MetS. Matsushita et al. (2015) showed that when the WC is in the ≥90th percentile, Japanese children (aged 10–12 years) are prone to having MetS. Highly intensive interval training is directly associated with balancing HbA1c and blood glucose levels (Pedrosa et al., 2022). Therefore, it can be assumed that engaging in daily high‐intensity interval training (Nishizawa, Akaoka, Nishida, Kawaguchi, Hayashi, & Yoshimura, 1976) might be associated with a lower risk of MetS in sumo wrestlers until their retirement. However, there are no further studies on sumo children or normal children that show an association between PhA and metabolic parameters, including HbA1c. The evaluation of PhA as an independent predictor of various clinical biochemical characteristics of MetS is gaining attention because of the compelling evidence presented in recent studies. Yucel et al. (2023) highlighted the significance of PhA as a potential marker reflecting metabolic status. Their research found a significant negative correlation between HbA1c and blood sugar levels and the value of all body segment PhA in T2DM patients. These findings suggest that an impaired glycemic index may negatively impact the integrity and health of cellular receptors. Furthermore, Praget‐Bracamontes et al. (2023) reinforced the role of PhA by showing its association with key aspects of MetS, including obesity, hyperuricemia, and vitamin D deficiency. This study emphasizes that low PhA values are correlated with worse outcomes in metabolic diseases. Thus, PhA could serve as a valuable non‐invasive and cost‐effective screening tool in clinical settings, offering a measure to evaluate the risk of MetS. Some studies (Barrea et al., 2016; Choi et al., 2020; Dittmar et al., 2015; Longo et al., 2021; Moreto et al., 2017) have shown an association between PhA and metabolic parameters including HbA1c in normal adults and in clinically arrested populations. PhA is negatively associated with HbA1c levels in patients with T2D (Choi et al., 2020; Dittmar et al., 2015), which may be due to increased blood glucose levels. Diabetes is directly associated with damage to body parts as well as body cells, resulting in the inhibition of cell integrity and cell capacity, which causes a decrease in PhA (Daryabor et al., 2020). However, our study participants were physically fit, and their HbA1c level was negatively associated with PhA.

A population‐based study conducted on adult males and females showed that an increase in WC is likely to decrease the PhA value (Longo et al., 2021). In our study, PhA showed a significant negative relationship with WC before accounting for age and weight. This pattern aligns with that of patients diagnosed with T2D, in which a reduction in the PhA value is linked to elevated HbA1c levels (Choi et al., 2020; Dittmar et al., 2015). Surprisingly, in our current investigation, PhA exhibited an inverse relationship with HbA1c, although our study participants were not affected by diabetes. Notably, HbA1c level serves as an additional indicator of T2D mellitus and a risk factor for cardiovascular disease (Sherwani et al., 2016). Onal et al. (2014) reported that increased HbA1c levels in children with obesity could be a critical factor contributing to insulin resistance and sensitivity. Furthermore, among patients with diabetes, high HbA1c levels are directly associated with a high risk of stroke, cardiovascular disease, and atrial fibrillation (Lu et al., 2015; Martín‐Timón, 2014; Portugal et al., 2020). HbA1c is not only an indicator of glycemic control, but also acts as a predictive marker for lipid profile levels. Elevated HbA1c concentrations are associated with an increase in cholesterol, TG, and LDL‐C, and a decrease in HDL‐C in patients with diabetes (Khan et al., 2007). HDL‐C levels in junior sumo wrestlers were significantly (*p* = 0.033) lower than the reference value and this value decreased as the age of the sumo wrestlers increased (Ogawa et al., 2021). Factors contributing to the reduction in HDL‐C levels include genetic factors, sedentary lifestyle, smoking, obesity, diabetes, and a diet enriched in refined carbohydrates such as white rice, sugar, and white bread (Khera & Plutzky, 2013). It can be assumed that the decline in HDL‐C levels observed in sumo wrestlers is linked to their dietary patterns and bodyweight. HDL‐C plays a pivotal role in the regulation of blood glucose levels through various mechanisms. Notably, it impedes pancreatic *ß*‐cell apoptosis and enhances insulin secretion. Additionally, HDL‐C curtails the overexpression of gluconeogenic hormones, thereby modulating blood glucose levels by inhibiting gluconeogenesis in the liver. Moreover, HDL‐C amplifies insulin receptors in muscle cells by activating GLUT‐4, which facilitates glucose uptake by these cells (van Linthout et al., 2010). Furthermore, HDL‐C and HbA1c levels demonstrate an inverse correlation, as reduced HDL‐C levels are associated with reduced glucose homeostasis (Huang et al., 2021; Maharjan et al., 2017). HbA1c originates from an irreversible Schiff base reaction in which glucose molecules bind to the N‐terminal end of the *β*‐globin chain of hemoglobin. Hydrogen peroxide (H_2_O_2_) promotes the release of ferrous iron (Fe^+2^) from HbA1c (Sen et al., 2005). H_2_O_2_ and Fe^+2^ together form Fenton's reagent, which undergoes the Fenton oxidation reaction, a key source of reactive oxygen species (ROS), such as OH^−^ (Chaudhury et al., 2017). Although ROS are essential for cell signaling, they also actively participate in cellular organelle damage (Auten & Davis, 2009) that disrupts fluid equilibrium between ICW and ECW. Such disruption can lead to an altered ECW/ICW ratio, adversely affecting the capacitive properties of the cell membranes. This in turn, led to a reduction in the PhA values (da Silva et al., 2023). Our study supports the notion that PhA is indeed a biomarker of altered metabolic status, which can be characterized by an apparent imbalance in fluid distribution, such as an increased ECW/ICW ratio and alteration of cell membrane structure and function. Furthermore, our findings suggest that PhA is associated with alterations in the cell membrane structure and function. This was confirmed by the significant negative relationship observed between PhA and HbA1c levels, suggesting that altered electrical properties of cells in fluids could underlie this association. Specifically, lower PhA values, which may reflect compromised cellular integrity, were associated with higher HbA1c levels, indicative of impaired glycemic control (*ß* = −0.496, *r* ^2^ = 0.776, *r* = −0.756, *p* = 0.004).

This study has certain limitations. Notably, the associations between PhA, metabolic parameters, and HbA1c levels were not analyzed in a control group. Additionally, the study did not include measurements of insulin levels and markers of oxidative stress and inflammation, nor did it assess the impact of variables such as energy expenditure, training intensity, and macronutrient intake on MetS components, fluctuations in HbA1c levels, and PhA values within the cohort of children studied. Furthermore, it is imperative to understand how adult sumo wrestlers are protected from MetS. Therefore, further research is essential to address these limitations and to enhance our understanding of these critical areas.

## 5 CONCLUSION

Our findings demonstrate that, contrary to metabolic parameters, HbA1c exhibits a negative correlation with PhA in junior sumo wrestlers, even after adjusting for age and weight. Based on the results of this study, we can assert that PhA is a potential predictive marker of glycemic control in junior sumo wrestlers.

## FUNDING INFORMATION

This study was supported by JSPS KAKENHI for KW (23K10738), HS (23H03279 and 23KK0177), and the Casio Science Promotion Foundation.

## CONFLICT OF INTEREST STATEMENT

None to declare.

## ETHICS STATEMENT

All participants signed a written informed consent form, and the protocol was approved by the Institutional Review Board of the Faculty of Health and Sports Sciences of University of Tsukuba (Ref No Tai 019–156).

## Data Availability

The datasets generated and/or analyzed in the current study are available from the corresponding author upon reasonable request.
